# Demographic Characteristics and Disease Pattern Among Non-COVID-19 Patients Admitted to Medical Units of a Tertiary Care Hospital in Sri Lanka During Lockdown of the COVID-19 Pandemic: A Comparison During and Before Lockdown

**DOI:** 10.7759/cureus.45248

**Published:** 2023-09-14

**Authors:** Mayurathan Pakkiyaretnam, Vaithehi R Francis, George Rajeevan Francis, Sanooz Raheem, Rajavarthani Sanjeev, Rajeswaran Rajavarman, Ramanathan Ramesh

**Affiliations:** 1 Department of Clinical Sciences, Faculty of Health-Care Sciences, Eastern University of Sri Lanka, Batticaloa, LKA; 2 University Medical Unit, Teaching Hospital Batticaloa, Batticaloa, LKA; 3 Department of Pathophysiology, Faculty of Health-Care Sciences, Eastern University of Sri Lanka, Batticaloa, LKA; 4 Microbiology, Teaching Hospital Batticaloa, Batticaloa, LKA; 5 Cardiology, Teaching Hospital Batticaloa, Batticaloa, LKA; 6 Department of Human Biology, Faculty of Health-Care Sciences, Eastern University of Sri Lanka, Batticaloa, LKA; 7 Emergency Medicine, Teaching Hospital Batticaloa, Batticaloa, LKA; 8 General Internal Medicine, Teaching Hospital Batticaloa, Batticaloa, LKA

**Keywords:** teaching hospital batticaloa, medical units, lockdown, disease pattern, demographic characteristics, covid-19

## Abstract

Background: The COVID-19 infection was rapidly spreading almost all over the world, and the first case was confirmed on 27th January 2020 by a foreign tourist in Sri Lanka. The first Sri Lankan citizen with COVID-19 was confirmed on 11th March 2020. Soon after the confirmation of the disease, long days of lockdown were imposed in almost all parts of the world, including Sri Lanka, to control the spread of the disease.

Objectives: To determine the demographic characteristics such as age, sex, number of patients, and disease pattern among non-COVID-19 patients admitted to the medical units during the lockdown of the COVID-19 pandemic and to compare these characteristics with the data before the lockdown.

Methods: This was a cross-sectional analytical study. It was conducted at the Teaching Hospital in Batticaloa, Sri Lanka. All the non-COVID-19 patients admitted to medical wards and intensive care units (ICU) were included in this study. Patients admitted to the medical intensive care unit (MICU) and coronary care unit (CCU) were considered ICU admissions in this study. They were studied over a period of one month during lockdown (11th March 2020 to 10th April 2020) and compared with the patients admitted one month prior to the lockdown (11th February 2020 to 10th March 2020).

Results: Totally, 2340 non-COVID-19 patients (52.5% males) were admitted before the lockdown, and 1376 non-COVID-19 patients (56.2% males) were admitted during the lockdown. This reduction in admission is statistically significant (p-value is <0.001, df=3715). Patients admitted to the wards before lockdown were 2283 (97.6%) and during lockdown were 1309 (95.1%). ICU admissions were N=57 (2.4%) before lockdown and N=67 (4.9%) during lockdown. The common age distribution before the lockdown showed that 26.4% were 31-50 and 41.5% were 51-70 years. Similarly, during lockdown, the age distribution disclosed that 28.9% were 31-50 years and 42.9% were 51-70 years. The disease pattern demonstrated that before lockdown, the majority of patients were admitted for routine hemodialysis (13.2%), to get an injection (9.9%), ischemic heart disease (8.4%), chronic kidney disease (7.3%), and viral fever, including dengue (7.2%). Likewise, during lockdown, more patients were admitted for routine hemodialysis (10.7%), viral fever, including dengue (9.3%), ischemic heart disease (8.8%), to get an injection (8.5%), and chronic kidney disease (5.9%).

Conclusion: There was a significant reduction in the number of non-COVID-19-related admissions during the period of lockdown. However, there was not much difference in the proportion of admissions according to gender, age, and disease pattern before and during lockdown. More number of male patients were admitted than female patients. Most of the admitted patients were under the age group of 51-70 years. The highest number of patients were admitted for routine hemodialysis before and during lockdown. However, a slightly higher number of patients were admitted to the ICU during lockdown. Therefore, strengthening the ICU facilities may be an important preparation to accommodate more patients in the future if a similar kind of emergency lockdown occurs in a pandemic situation. In addition, admissions due to non-communicable diseases (NCD) didn't fall in proportion during the pre-COVID-19 period and the lockdown period. Therefore, the redistribution of healthcare facilities needs to be done wisely to face the challenges caused by the NCDs.

## Introduction

COVID-19 is a newly discovered viral infection since December 2019. It was isolated first from a patient with pneumonia in Wuhan, China, and within a few weeks, the disease was spreading almost all over the world as a pandemic [1]. The disease is caused by an RNA virus, which is named severe acute respiratory syndrome coronavirus-2 (SARS‑CoV‑2). The virus primarily spreads by respiratory droplets when an infected person coughs, sneezes, speaks, sings, or even breathes [2]. Normally majorities (80%) of people are asymptomatic or develop mild to moderate respiratory illness. However, older people with underlying medical problems such as cardiovascular disease, diabetes mellitus, chronic lung diseases, and malignancies are more likely to develop critical illness, and eventually 1-3% will die [3].

The first case of COVID-19 infection was confirmed on 27th January 2020 by a foreign tourist in Sri Lanka. The first Sri Lankan citizen with COVID-19 was confirmed on 11th March 2020 [4,5]. As of 31st July 2023, there had been 768,559,963 confirmed cases and 6,952,509 deaths due to COVID-19 globally. In Sri Lanka, 672,564 confirmed cases and 16,880 deaths have been reported [6].

As this pandemic spreads rapidly all over the world, lockdown and curfews were imposed in almost all parts of the world including Sri Lanka. The first lockdown was imposed in Sri Lanka on 11th March 2020 following the confirmation of the first Sri Lankan patient with COVID-19 [5]. In addition, people were instructed to stay at home and practice good hygienic measures such as one-meter social distancing, frequently washing hands with soap and water or 70% alcohol hand sanitizers, and wearing face masks [1].

Subsequently, more and more COVID-19 cases were confirmed, which required more admissions to the hospitals. Therefore, most of the teaching hospitals in Sri Lanka have been recognized as treatment centers for COVID-19, including the Teaching Hospital in Batticaloa. It was obvious that a massive number of COVID-19 cases were admitted to health institutions daily, and on the other hand, the number of patients admitted with other medical problems was significantly reduced. This led to presentations with severe diseases and a late presentation on admission. As a result, the heads of the institutions and policymakers were struggling to modify the hospitals within a short period of time, considering the requirements based on COVID-19. This caused a challenge in the routine healthcare services provided in Sri Lanka.

The findings from other parts of the world regarding the number of non-COVID-19 admissions during the COVID-19 lockdown period showed a reduction compared to the pre-COVID-19 data. The reduction was statistically significant, and various factors such as the limitations in transport, the redistribution of healthcare facilities, and the reduction in cases of other communicable diseases due to distancing and better hygienic practices were attributed as the reasons [7-10]. A study conducted at the Teaching Hospital, Karapitiya, in southern Sri Lanka revealed a reduction in the number of cases admitted to the medical wards and the emergency treatment units (ETU) during the COVID-19 lockdown. This study highlighted that there was no reduction in the proportion of cases with non-communicable diseases (NCDs) [11]. Interestingly, worldwide studies have shown that there was a reduction in the number of pediatric emergencies, mainly those due to trauma, and the number of consultations with general physicians during the COVID-19 lockdown [12,13].

Similar studies of this nature from other parts of Sri Lanka are lacking. The Teaching Hospital, Batticaloa, serves as a specialized care center providing coverage for the Eastern, North-Central parts of Sri Lanka, and a similar study will help to plan the distribution of health facilities in any pandemics or emergencies.

The main aim of the study was to compare the demographic characteristics and disease patterns among non-COVID-19 patients admitted to medical units of Teaching Hospital, Batticaloa, Sri Lanka, during the lockdown of the COVID-19 pandemic with the pre-COVID-19 period.

## Materials and methods

The aim of this study was to address the demographic patterns and distribution of medical problems during the first lockdown period in Sri Lanka. The data collected during the COVID-19 lockdown period were compared with similar data before the COVID-19 lockdown. The specific objectives of the study were to determine the demographic characteristics of patients, such as the age, sex, number of patients, and disease pattern among those patients admitted to medical units of Teaching Hospital, Batticaloa during the lockdown of the COVID-19 pandemic and to compare these characteristics with the available data before the lockdown period. The study setting included medical units such as medical wards and intensive care units (ICUs). Patients admitted to the medical intensive care unit (MICU) and coronary care unit (CCU) were considered ICU admissions in this study.

This cross-sectional retrospective analytical study was conducted among patients admitted to medical wards and intensive care units of the Teaching Hospital, Batticaloa during the COVID-19 pandemic and the pre-COVID-19 period. The study was conducted over a period of one month during lockdown (11th March 2020 to 10th April 10), and the data were compared with those of the patients admitted one month prior to lockdown (11th February 2020 to 10th March 2020). Data related to non-COVID-19 admission was collected from the hospital records available at the medical wards, MICUs, and CCUs. Age, gender, date of admission, and diagnosis were collected as the baseline data from 11th March 2020 to 10th April 2020. Similar pre-COVID-19 data was collected from 11th February 2020 to 10th March 2020. This study only included admissions to the medical wards, MICUs, and CCUs with underlying medical causes for admissions. It didn’t include COVID-19 patients and patients admitted under pediatric, surgical, psychiatric, obstetric and gynecological, oncological, and other categories.

Ethical approval (E/2020/09) for this study was obtained from the Ethics Review Committee, Faculty of Health-Care Sciences, Eastern University, Sri Lanka. There was no direct human interaction while collecting the data, and only the relevant details related to the study were collected from the records.

The data was entered and analyzed in SPSS version 25 (IBM, Inc., Chicago, IL, USA). A P-value of ≤0.05 was considered as statistically significant. 

## Results

Total admission for one month before the lockdown period was 2340 (52.5% males; n=1228 and 47.5% females; n=1112) and during the one month of lockdown, it was 1376 (56.2% males; n=773 and 43.8% females; n=603). The number of admissions showed a 41.19% reduction from the pre-COVID-19 period to the COVID-19 lockdown. This reduction in admission was statistically significant (p-value <0.001). However, there was no statistically significant difference in the proportion of admitted male and female patients before and during lockdown.

**Figure 1:**
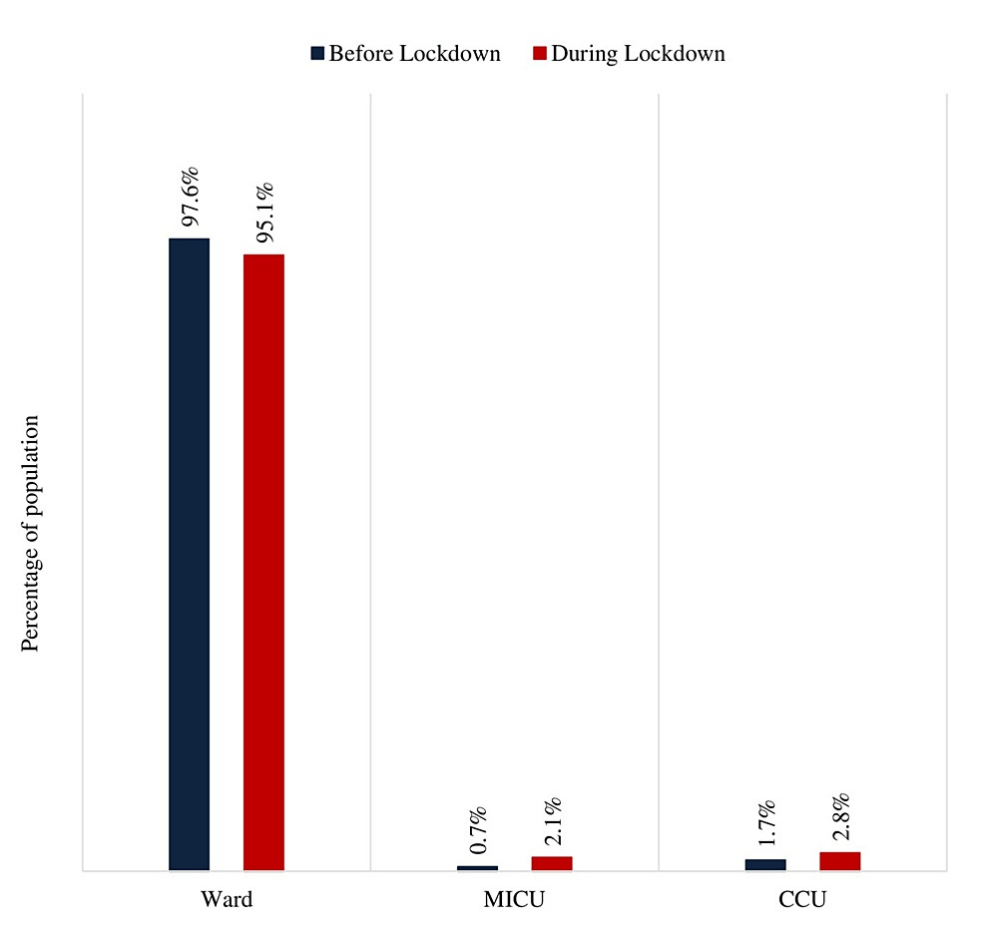
Proportion of the patient population according to the place of admission before and during lockdown. MICU: medical intensive care unit, CCU: coronary care unit.

Patients admitted to the medical wards before the lockdown were 2283 (97.6%), and during the lockdown were 1309 (95.1%). Total ICU admissions were 57 (2.4%) before lockdown and 67 (4.9%) during lockdown. There was no statistically significant difference in admissions to wards or ICUs before and during lockdown (Figure 1).

The age distribution showed more patients were between 51 and 70 years old before and during the lockdown. However, these age groups did not show any statistically significant differences (Table 1). However, admissions within the age group of 14-20 years old decreased significantly.

**Table 1:** Age distribution among admitted patients before and during lockdown.

Age category (in years)	Before lockdown	During lockdown	P-value
14-20	223 (9.5%)	97 (7%)	0.0086
21-30	265 (11.3%)	152 (11%)	0.7794
31-40	241 (10.3%)	148 (10.8%)	0.6310
41-50	377 (16.1%)	249 (18.1%)	0.1157
51-60	496 (21.2%)	289 (21%)	0.8853
61-70	474 (20.3%)	301 (21.9%)	0.2467
71-80	212 (9.1%)	118 (8.6%)	0.6056
81-90	49 (2.1%)	22 (1.6%)	0.2829
91-100	3 (0.1%)	0	0.2407

**Figure 2:**
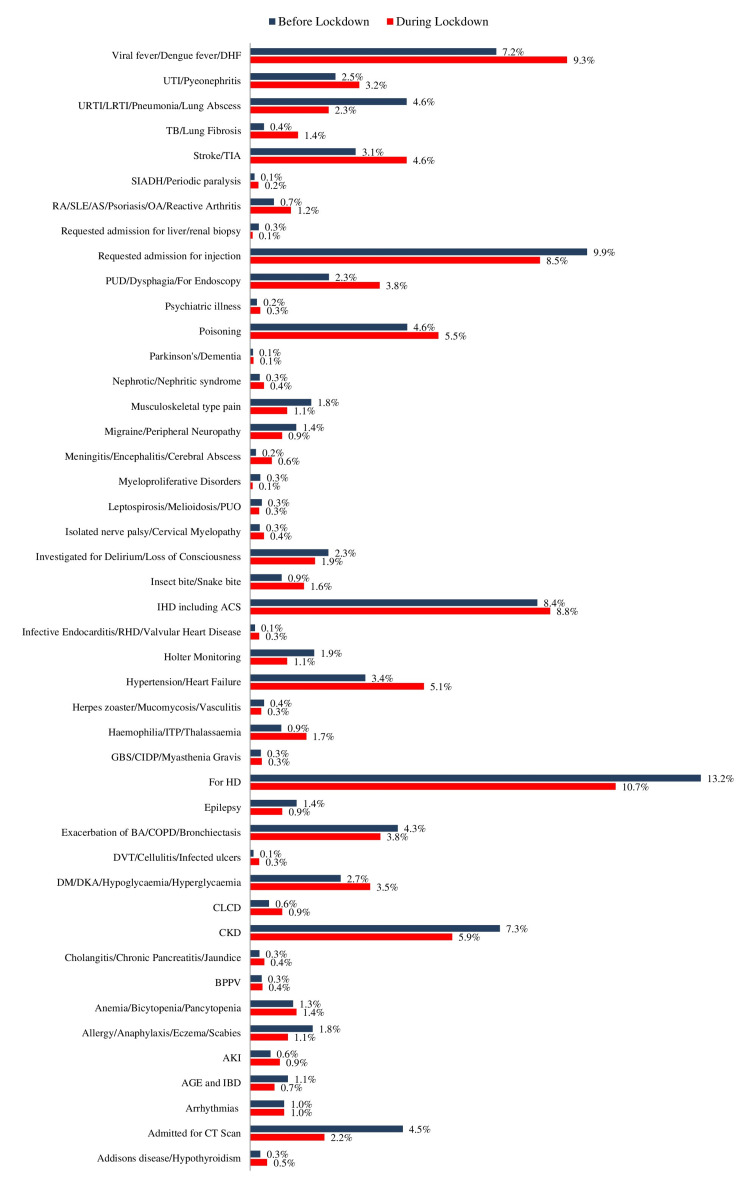
Pattern of disease distribution among admitted patients before and during lockdown. DHF: dengue hemorrhagic fever, UTI: urinary tract infection, URTI: upper respiratory tract infection, LRTI: lower respiratory tract infection, TB: tuberculosis, TIA: transient ischemic attack, SIADH: syndrome of inappropriate anti-diuretic hormone (ADH) secretion, RA: rheumatoid arthritis, SLE: systemic lupus erythematosus, AS: ankylosing spondylitis, OA: osteoarthritis, PUD: peptic ulcer disease, PUO: pyrexia of unknown origin, IHD: Ischemic heart disease, ACS: acute coronary syndrome, RHD: rheumatic heart disease, ITP: idiopathic thrombocytopenic purpura, GBS: Guillain-Barré syndrome, CIDP: chronic inflammatory demyelinating polyneuropathy, HD: hemodialysis, BA: bronchial asthma, COPD: chronic obstructive pulmonary disease, DVT: deep vein thrombosis, DM: diabetes mellitus, DKA: diabetic ketoacidosis, CLCD: chronic liver cell disease, CKD: chronic kidney disease, BPPV: benign paroxysmal positional vertigo, AKI: acute kidney injury, AGE: acute gastroenteritis, and IBD: inflammatory bowel disease.

Disease pattern demonstrated that, before lockdown, majority of patients were admitted for routine hemodialysis (HD) (13.2%), to get an injection (9.9%), ischemic heart disease (IHD) including acute coronary syndromes (ACS) (8.4%), chronic kidney disease (CKD) (7.3%), and viral fever including dengue fever (DF) and dengue hemorrhagic fever (DHF) (7.2%). Likewise, during the lockdown, most of the patients were admitted for routine HD (10.7%), viral fever including DF and DHF (9.3%), IHD including ACS (8.8%), to get an injection (8.5%), and CKD (5.9%). The study did not reveal any significant difference in the proportion of admissions based on the disease pattern (Figure 2).

## Discussion

As expected, the number of patients admitted for medical reasons (except COVID-19) during lockdown was reduced, which was statistically significant in this study. The reduction was nearly 41% in this study, and a study carried out in the US showed a similar reduction (43%) [14]. Even though the number of patients admitted was reduced, there were not many differences in gender or age distribution except in the 14-20-year-old group between pre-COVID-19 and lockdown data. It was observed that a number of male patients and in between the age of 51-70 years were mostly admitted before and during lockdown. Disease distribution also did not show any statistically significant changes in the proportion of admissions during lockdown and pre-COVID-19 period. Interestingly, more patients were admitted for hemodialysis before and during lockdown. The necessity of regular hemodialysis would have been the contributory factor for this finding. The study revealed that patients admitted to the ICUs was slightly higher during lockdown when compared to before lockdown. It could be due to delay in admission leading to more severe disease. However, this increase in ICU admission was not statistically significant.

The findings of this study are very similar to the findings from the study carried out at Teaching Hospital, Karapitiya in Sri Lanka [11]. The reduction in the number of admissions in both hospital settings has been statistically significant and the distribution of diseases among the admitted patients has been almost similar in the cases of NCDs. Chronic kidney disease, IHD with ACS, and other NCDs have been the major causes of admissions. While infective diseases such as other respiratory infections, dengue, and DHF have reduced in number at Karapitiya Hospital, there has been no significant reduction in the proportion of these cases at Batticaloa. The monsoon rain and other adverse weather conditions might have been the reason for this difference. Similarly, there has been a decrease in non-COVID-19 admissions in countries like Germany, Denmark, Italy, Korea, the UK, the USA, and China [7,14-19]. The majority of admissions at these settings have been due to IHD and stroke; in other terms, the NCDs have been the major cause. A study carried out in a rural healthcare setting in South Africa showed no association between lockdown and all-cause daily admissions [8]. However, the admission of children under five years old has been decreasing at various levels of lockdown in South Africa. In our study, the number of admissions among young adults (14-20 years old) was significantly low. This may be due to better hygienic practices and social distancing, which reduce the possibility of getting other types of infections, such as respiratory tract infections and food-borne infections. A study carried out in Belgium [9] showed a reduction in the total number of admissions, while the distribution of disease among the admitted patients showed a greater number of cases with NCDs, where the number of cancer patients didn’t drop during the lockdown. In our study, we didn’t include patients with cancer, but as noted in our findings, IHD and other NCDs have been showing a similar pattern as in the study carried out in Belgium [9]. Another study carried out in Australia [10] showed that there was a significant reduction in the number of admissions, and the important feature of this study was that they compared the lockdown data with the data belonging to the same period for the previous three years. This showed a significant reduction in the number of admissions and confirmed that the daily admissions due to IHDs didn’t change during the lockdown.

The findings from our study show the same pattern in the reduction of non-COVID-19 admissions according to the data available from the international and local study findings. There can be various factors contributing to the reduction in the number of admissions. In our study, Teaching Hospital Batticaloa is providing the service for a larger geographical region as the Teaching Hospital with specialized facilities. It receives transfers from peripheral centers, and patients travel more than 100 miles to obtain the services. The lockdown created a challenge in traveling distances, and this would have contributed to patients’ access to this healthcare facility. Similarly, patients would have accessed the closest peripheral facilities rather than facing difficulties in traveling long distances to seek service at a specialized center such as Batticaloa. The introduction of strong hygienic practices, social distancing, and various other remedies might have caused a reduction in other forms of communicable diseases. A study carried out in China attributed the behavioral changes among the patients and healthcare providers during COVID-19, the suspension of non-emergency health facilities, the ban on mobility, and the reduction in non-COVID-19 diseases as the main reasons for this observation [18].

Moreover, the important aspects of this study reiterate that the healthcare facility for NCDs should be adequate to meet the patients’ demands. The number of people with NCDs is increasing day by day, and this causes a bigger challenge in healthcare emergencies, such as pandemics. However, the NCDs are becoming a challenge for the allocation or redistribution of healthcare facilities. As described by Smith et al., the priority settings in healthcare resource allocation should focus on NCDs [20]. Facilities for dialysis, management of IHD, and other complications of NCDs should be available adequately. It is noteworthy that the number of critical/ICU admissions increased during the lockdown period, as per the findings from our study. This would be due to the delay in seeking healthcare facilities caused by the lockdown and the travel restrictions. However, this increased the requirement for ICU admissions.

The mobility of healthcare resources should be done considering all these demographic factors and disease patterns. Reservation of ICU beds or other facilities in cases of health emergencies or pandemics should address the challenge caused by the NCDs. An adequate facility needs to be made available for the healthcare conditions caused by NCDs. This is not a unique situation for the Teaching Hospital, Batticaloa. Studies have signified the implication of NCDs during pandemic situations [15,21].

This study signifies the following as the important findings; that the number of routine admissions can reduce in cases of pandemics. The other emergencies caused by medical or NCDs would require enough ICU facilities to handle the pandemic effectively. Studies in other settings have identified a significant reduction in the number of emergency admissions due to trauma [12] and consultations at general practice services [13]. These factors can be taken into consideration while preparing emergency health response plans during lockdowns or pandemics. The preparation of an emergency health plan response can be done based on local findings addressing demographic factors and disease patterns. Our study has shown the changes occurring in the pattern of admission during the pandemic, which is almost similar to the international context, and has addressed the variations in the disease patterns that can be specific to geographical areas, even within a country. Our finding adds more value to the statement in planning health care response during pandemics as stated by Collins et al. “Collaborative multisectoral and multistakeholder governance is critical to respond to emerging health challenges, including the NCD and Communicable Diseases (CD) syndemic, and the underlying social, economic and environmental determinants” [22].

Including admissions to the medical units and ICUs (MICU and CCU) were the major limitations of this study. In addition, this was conducted retrospectively and only carried out during the initial lockdown period. Further studies would have been carried out during the other waves of COVID-19, and those findings would have given more input for the findings in this study. The collected data during the lockdown period was compared only with the data of the previous month, pre-COVID-19. Similarly, the utilization of healthcare facilities at the peripheral centers could have been studied to see whether the fall in admissions at this healthcare facility was affected by the increased utilization of the peripheries.

## Conclusions

There was a statistically significant reduction in the number of admissions during the period of lockdown. However, there was not much difference in the proportion of admissions according to gender, age, or disease pattern. Gender and age revealed that more patients were males under the age group of 51-70 years admitted before and during lockdown. Younger adults (14-20 years old) showed a significant reduction in admissions. The highest number of patients admitted for routine hemodialysis before and during lockdown. However, a slightly higher number of patients were admitted to the ICU during lockdown. NCDs can become a challenge while handling similar kinds of health emergencies and pandemics. We recommend that strengthening the ICU facilities may be important to accommodate more patients in the future if a similar kind of emergency lockdown occurs and the redistribution of healthcare facilities needs to be done wisely to face the challenges caused by the NCDs. Moreover, similar large-scale studies need to be conducted, including all the specialties and peripheral centers, to see whether the fall in admissions at this healthcare facility was affected by the increased utilization of peripheries.
